# Molecular genetic studies on EGFR, KRAS, BRAF, ALK, PIK3CA, PDGFRA, and DDR2 in primary pulmonary adenoid cystic carcinoma

**DOI:** 10.1186/s13000-015-0409-7

**Published:** 2015-09-15

**Authors:** Zhen Huo, Huanwen Wu, Shanqing Li, Zhiyong Liang

**Affiliations:** Department of Pathology, Peking Union Medical College Hospital, Chinese Academy of Medical Sciences & Peking Union Medical College, No. 1 Shuaifuyuan, Wangfujing Street, Dongcheng District, Beijing, 100730 China; Department of Thoracic Surgery, Peking Union Medical College Hospital, Chinese Academy of Medical Sciences & Peking Union Medical College, Beijing, 100730 China

## Abstract

**Background:**

Pulmonary adenoid cystic carcinoma (PACC) is an uncommon neoplasm of the lung but represents the predominant type of salivary gland-type lung carcinoma. Only a few studies have focused on the genetic events associated with PACC. The aim of this study was to characterize the genetic events associated with PACC.

**Findings:**

We reviewed 24 cases of primary PACC between 2000 and 2014, including 21 cases reported in our previous study and three new cases added in 2014. Mutation profiling of the EGFR, KRAS, BRAF, ALK, PIK3CA, PDGFRA, and DDR2 genes was performed using next-generation sequencing, Sanger sequencing, and quantitative polymerase chain reaction in 9 successfully amplified cases. The 24 cases of PACC included 7 men and 17 women, aged 24–74 years (mean, 50.8 years). All the cases were located in the trachea or bronchus. No mutations were detected in any of the seven genes in the nine cases that qualified for mutation analysis, and the results using different methods were consistent.

**Conclusions:**

The data presented in this work suggest that EGFR, KRAS, BRAF, ALK, PIK3CA, PDGFRA, and DDR2 may not be driver genes in primary pulmonary adenoid cystic carcinoma.

## Findings

### Introduction

Primary pulmonary adenoid cystic carcinoma (PACC) is a rare neoplasm. It is presumed to originate from the minor salivary glands lining the tracheobronchial tree and is one of the main types of salivary gland-type carcinoma of the lung [1]. Although many molecular genetic studies have implicated certain genetic mutations in non-small cell lung cancer (NSCLC), including mutations in the EGFR, PIK3CA, BRAF, KRAS, and ALK genes [2, 3], only a few studies have focused on the genetic events associated with salivary gland-type lung carcinomas. With the exception of the recent discovery of translocations and fusion oncogenes in salivary gland tumours, a few studies have reported that genetic alterations in genes such as EGFR, KIT, BRAF, CCND1, HRAS, KRAS, NRAS, PIK3CA, and PDGFRA occur in malignant salivary gland tumours at a lower frequency [4–16]. Gene alterations in KIT, EGFR, BRAF, HRAS, KRAS, NRAS, PIK3CA, PDGFRA, and PTEN have been reported in adenoid cystic carcinoma (ACC) [4, 5, 7–16], but the results are inconsistent among different studies [10, 12, 17]. The genetic studies of PACC are scarce, and no genetic alterations, such as in EGFR and KIT, have been detected in these studies [18, 19]. In the current study, we reviewed a retrospective series of 24 patients with primary PACC and evaluated the EGFR, KRAS, BRAF, ALK, PIK3CA, PDGFRA, and DDR2 gene status using three different methods, including next-generation sequencing (NGS), Sanger sequencing, and quantitative polymerase chain reaction (QPCR).

## Materials and methods

### Patients and specimens

We reviewed all the surgical lung biopsy or resection records at Peking Union Medical College Hospital from 2000 to 2014 and identified a total of 24 cases of PACC, including 21 cases reported in our previous study [20] and three new cases added in 2014. No patient had a history of a salivary gland tumour. All the samples were fixed in 10 % neutral buffered formalin, routinely processed, and embedded in paraffin. Haematoxylin-eosin-stained sections were observed by optical microscopy and reviewed independently by three experienced pathologists based on the World Health Organization criteria for PACC [1]. The ethics committee of Peking Union Medical Collage Hospital specifically approved this study, and informed consent was obtained from all patients.

Genomic DNA from 21 PACC samples with sufficient available tissue was extracted from freshly cut formalin-fixed, paraffin-embedded tissue sections using a QIAamp DNA Mini Kit (Qiagen, Germany) according to the manufacturer’s instructions. The tumour area was identified through haematoxylin-eosin staining, and tissue from this area on unstained sections was removed for DNA extraction. The extracted DNA was then quantified using the Qubit dsDNA BR Assay (Life Technologies, USA). Out of 21 cases of PACC, DNA from nine cases was successfully amplified. Mutational analysis was performed using three different methods, including NGS, Sanger sequencing, and QPCR.

### NGS and data processing

Targeted NGS was performed with 10 ng of DNA as the template to generate the amplicon library for sequencing. Libraries were prepared using the Ion AmpliSeq Library Kit 2.0 (Life Technologies, USA) and the Lung Cancer Mutation Panel (ACCB Biotech, China), which is designed to detect mutations within 16 exons of seven lung cancer driver genes (EGFR, KRAS, BRAF, ALK, PIK3CA, PDGFRA, and DDR2) (Table 1). Adapter ligation, nick repair, and PCR amplification were performed according to the manufacturer’s protocol. Libraries were then quantified using a Qubit dsDNA HS Assay Kit and a Qubit 2.0 fluorometer (Life Technologies, USA), with samples diluted to a concentration of 3 ng/mL and pooled in equal volumes. Emulsion PCR and enrichment steps were performed using an Ion OneTouch Template Kit on the Ion OneTouch system (Life Technologies, USA) according to the manufacturer’s protocol. After enrichment, the amplicon libraries were subjected to sequencing on the Ion Torrent PGM system (Life Technologies, USA) using 318 chips and barcoding with the Ion Xpress Barcode Adapters 1–16 Kit (Life Technologies, USA). After sequencing, reads were mapped to the reference genome (hg19) using the Torrent Mapping Alignment Program (TMAP). Variants were identified using Torrent Variant Caller (version 3.6.6; Life Technologies, USA). The Integrative Genomics Viewer (Broad Institute, USA) was used to visualize variants against the reference genome to confirm the accuracy of the variant calls by checking for possible strand biases and sequencing errors.

**Table 1:** The 16 exons of the seven genes analysed in the present study

Genes	Exons
EGFR	Exons 18, 19, 20 and 21
KRAS	Exons 2 and 3
BRAF	Exons 11 and 15
PIK3CA	Exons 9 and 20
ALK	Exons 23 and 25
DDR2	Exon 18
PDGFRA	Exons 12, 14 and 18

### Sanger sequencing

Mutations within 16 exons of the seven lung cancer driver genes were also screened by PCR-based bidirectional direct Sanger sequencing using primers. The sequencing results were interpreted using Chromas software version 1.45 (Technelysium Pty, Australia).

### QPCR

The Human Mutation Qualitative Detection Kit (ACCB Biotech, China) was used according to the manufacturer’s instructions. QPCR was performed on a Rotor-Gene QPCR Platform (Qiagen, Germany). The cycling conditions for quality control (QC) runs and for mutation assays were as follows: 10 min at 95 °C followed by 40 cycles of 95 °C for 15 s and 60 °C for 1 min. Fluorescence was measured at 60 °C. Data regarding each mutation were interpreted according to the kit manual after curve analysis and calculation of ΔCt values.

## Results

**Fig. 1:**
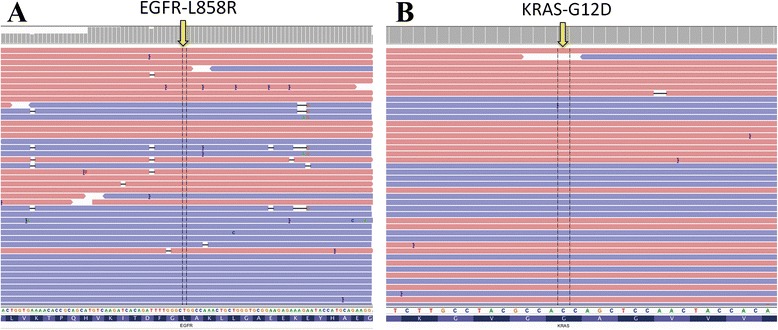
No mutations were found in PACC cases by NGS. **a** No L858R mutations were found in EGFR. **b** No G12D mutations were found in KRAS

**Fig. 2:**
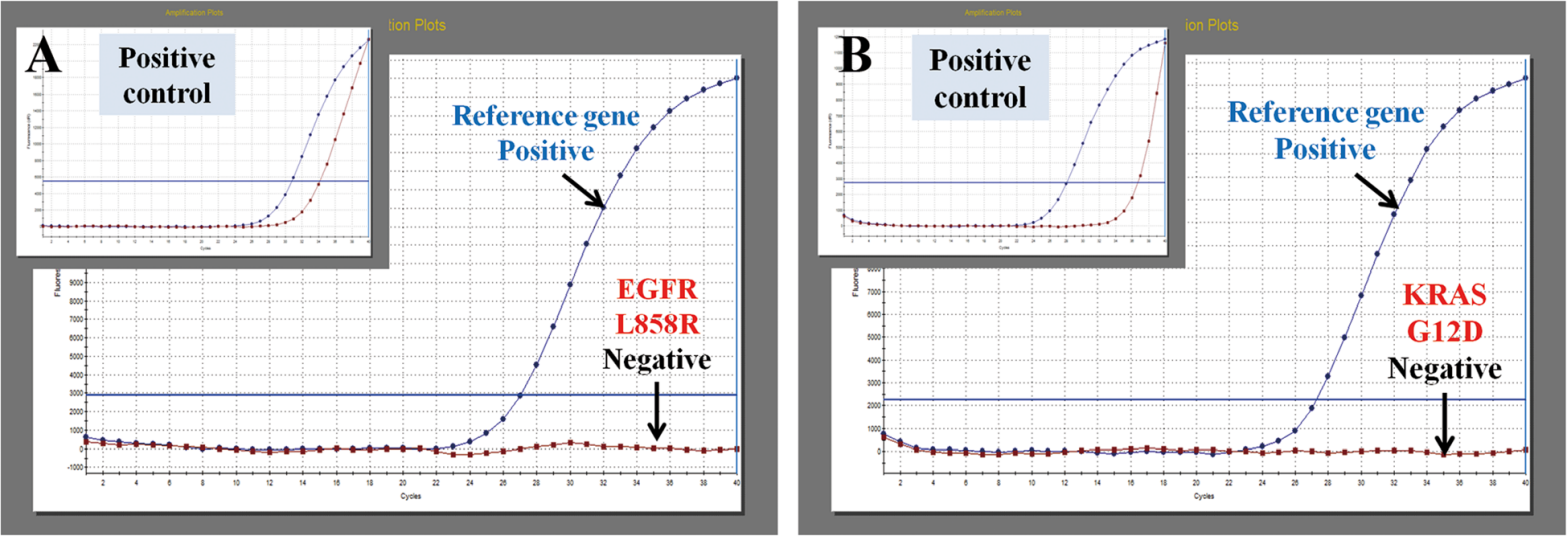
No mutations were found in PACC by QPCR (the same case as in Fig. 1). **a** No L858R mutations were found in EGFR. **b** No G12D mutations were found in KRAS

**Fig. 3:**
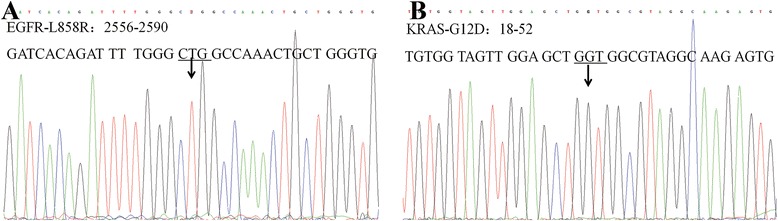
No mutations were found in PACC by Sanger sequencing (the same case as in Fig. 1). **a** No L858R mutations were found in EGFR. **b** No G12D mutations were found in KRAS

The 24 cases of PACC included 7 men and 17 women, with a mean age of 50.8 years, and accounted for 0.23 % of all 10500 cases of primary histologically diagnosed malignant pulmonary tumours. Five patients had a history of smoking. Cough, dyspnea, and haemoptysis were the most common symptoms. All 24 primary cases were located in the trachea or bronchus, and they all exhibited typical PACC histopathology. Follow-up data (range, 1–132 months) was available for 18 patients, one patient died of a surgical complication, and five patients experienced disease recurrence and/or metastasis. No mutations were found within 16 exons of the EGFR, KRAS, BRAF, ALK, PIK3CA, PDGFRA, and DDR2 genes using NGS, Sanger sequencing, and QPCR in 9 successfully amplified cases (Table 2, Figs. 1, 2, 3). The results using the three methods were consistent.

**Table 2:** Clinical and genetic data for the 9 successfully amplified cases with PACC

No.	Age (y)	Gender	Location	Genetic mutation						
				EGFR	KRAS	BRAF	PIK3CA	ALK	DDR2	PDGFRA
1	38	M	Trachea (upper 1/3)	-	-	-	-	-	-	-
2	56	F	Trachea (upper 1/3)	-	-	-	-	-	-	-
3	35	F	Trachea (lower 1/3)	-	-	-	-	-	-	-
4	60	F	Trachea (lower 1/3)	-	-	-	-	-	-	-
5	51	F	Trachea (lower 1/3)	-	-	-	-	-	-	-
6	57	F	Trachea (upper 1/3)	-	-	-	-	-	-	-
7	62	F	Trachea (middle 1/3)	-	-	-	-	-	-	-
8	74	F	Trachea (upper 1/3)	-	-	-	-	-	-	-
9	46	F	Trachea (lower 1/3)	-	-	-	-	-	-	-

## Discussion

Primary salivary gland-type tumours of the lung are rare [1] and differ from the more common types of lung cancer. As the main type of salivary gland-type lung carcinoma, PACC is difficult to diagnose and cure at the early stage and is unlikely to be completely surgically removed. Postoperative radiotherapy is helpful for reducing the likelihood of recurrence and metastasis [20]. However, only limited data are available on the role of conventional systemic and targeted therapies in the management of patients with advanced disease. There is perhaps a need to develop new molecular biomarkers to improve the therapeutic options for these patients. Recently, important advances have been made in ACC; a signature t(6;9)(q22–23; p23–24) chromosomal translocation resulting in a MYB–NFIB fusion gene was identified, and the fusion oncoprotein activates the transcription of MYB targets that are important for oncogenic transformation. An increasing number of studies has demonstrated that MYB activation occurs in more than 80 % of cases of ACC, including PACC [4]. In this study, we aimed to identify driver genes other than MYB in PACC.

Genetic alterations associated with the development of NSCLC have been extensively characterized. The driver genes involved in lung adenocarcinoma include KRAS, EGFR, ALK, and BRAF [2], and those implicated in lung squamous cell carcinoma (LSCC) include PIK3CA, FGFR1, EGFR, PDGFRA, and DDR2 [3]. However, the mutational status of these genes in PACC has not been well characterized. Activating mutations in EGFR identify those NSCLC patients with an improved clinical response to tyrosine kinase inhibitor (TKI) therapy, but it remains unknown whether patients with PACC harbour EGFR mutations and can thus benefit from TKI therapy. EGFR mutations have been reported in pulmonary and salivary mucoepidermoid carcinoma [21], but they are rare in ACC of the salivary gland [14, 15], and no EGFR mutations were detected in PACC in a previous study [18]. Similarly, in our series, no mutations in EGFR were detected. A few studies have identified alterations in KRAS in ACC [7, 12, 13], and KRAS alterations were reported to be more common than other gene alterations, with the exception of MYB, in a recent study [12]; however, KRAS mutations were absent in other studies that involved whole exome sequencing of ACC [16] and next-generation sequencing [15]. There are no relevant studies on KRAS in PACC in the literature, and KRAS mutations were not detected in our series. Genetic alterations in PIK3CA [8, 11, 15, 16] and BRAF [10, 13] have been detected in ACC at a lower frequency than KRAS, and a study suggested that the PI3K/AKT pathway may be responsible for the unusually aggressive course of ACC [8]. There are no similar relevant studies in PACC, and no PIK3CA and BRAF gene mutations were detected in our series. ALK gene alterations mainly occur in lung adenocarcinoma and are associated with gene rearrangements. ALK inhibitors exhibit marked anti-tumour activity against lung cancers with ALK rearrangements [22]. However, emerging genomic data are revealing common ALK point mutations in various cancer types other than lung cancer, and several recent studies have demonstrated that ALK point mutations, independent of ALK gene rearrangements, can be oncogenic [23]. Genetic alterations of DDR2 and PDGFRA are associated with the development of LSCC [3, 24, 25]. Recently, DDR2 mutations were reported in approximately 4 % of LSCC cases, and some of these mutations induced oncogenic transformation. DDR2 mutations are associated with increased sensitivity to dasatinib, and the clinical activity of dasatinib in lung cancer is being evaluated in numerous clinical trials [24]. Platelet-derived growth factor receptors (PDGFRs) and their ligands play critical roles in several human malignancies. Sunitinib is a clinically approved, multi-targeted TKI that inhibits PDGFR with demonstrated clinical activity in gastrointestinal stromal tumours. However, some rare tumours, including LSCC, that demonstrate PDGFRA activation may also be clinically responsive to pharmacologic PDGFRA inhibition [25]. However, alterations in the ALK and DDR2 genes have not yet been investigated in ACC, and PDGFRA was only detected in two ACC cases [15]. In our series, there were no mutations in the ALK, DDR2, and PDGFRA genes. Our study suggested that the genetic mutations associated with PACC are different from those implicated in NSCLC, and EGFR, KRAS, BRAF, ALK, PIK3CA, PDGFRA, and DDR2 might not be driver genes in PACC.

The results of the present study are important in that they indicate that the targeted gene therapies for NSCLC may be useless in patients with PACC because of the different gene profiles. Our study has some limitations. First, the number of samples in the study was relatively small because of the low incidence of PACC and the limited success in extracting DNA for amplification over a long time span of 15 years. Second, only seven genes were analysed using three methods. We will expand the scope of the genes and increase the number of cases by involving other institutions in our future study of PACC to identify other genes for targeted therapy against PACC. Interestingly, we performed mutation profiling using three methods, including NGS, Sanger sequencing, and QPCR, and the results were consistent. Although the gold standard methods for detecting gene mutations are Sanger sequencing and QPCR, we believe that NGS is as good as the other methods because similar results were obtained in our study and because NGS saves time and uses less sample tissue. To date, only one study on ACC has used NGS technology [15]; our study is the second to analyse genetic mutations in ACC using NGS methodology.

In conclusion, the genetic mutations associated with PACC are different from those implicated in non-small cell lung cancer, and EGFR, KRAS, BRAF, ALK, PIK3CA, PDGFRA, and DDR2 may not be driver genes in PACC; we must identify other genes for targeted therapy against PACC.

## Abbreviations

NGS: Next generation sequencing
NSCLC: Non-small cell lung cancer
LSCC: Lung squamous cell carcinoma
PACC: Pulmonary adenoid cystic carcinoma
PDGFR: Platelet-derived growth factor receptor
QPCR: Quantitative polymerase chain reaction
TKI: Tyrosine kinase inhibitor
